# Eotaxin and IL-4 levels are increased in induced sputum and correlate with sputum eosinophils in patients with nonasthmatic eosinophilic bronchitis

**DOI:** 10.1097/MD.0000000000006492

**Published:** 2017-03-31

**Authors:** Rui Zhang, Wei Luo, Zhenyu Liang, Yaxia Tan, Ruchong Chen, Wenju Lu, Nanshan Zhong

**Affiliations:** a State Key Laboratory of Respiratory Disease, National Clinical Research Center for Respiratory Disease, Guangzhou Institute of Respiratory Disease, First Affiliated Hospital of Guangzhou Medical University, Guangzhou Medical University, Guangzhou, Guangdong; b Department of Respiratory and Critical Care Medicine, First Affiliated Hospital of Zhengzhou University, Zhengzhou University, Zhengzhou, Henan, China.

**Keywords:** eotaxin, IL-4, inflammation profile, nonasthmatic eosinophilic bronchitis, sputum eosinophil percentage

## Abstract

Nonasthmatic eosinophilic bronchitis (NAEB) is characterized by chronic cough and airway eosinophilic inflammation. Airway and systemic inflammation cytokine profile have not been comprehensively described in patients with NAEB.

The aim of the study was to identify the cytokine profile in sputum and serum of NAEB patients. Furthermore, the relationship between cytokines and clinical features would be evaluated.

Induced sputum and serum were collected from untreated NAEB patients and healthy subjects. The cytokine profile in sputum and serum was analyzed by a bead-based multiplex cytokine assay including 21 cytokines.

The levels of EGF, eotaxin, GM-CSF, GRO, IFN-γ, IL-1β, IL-4, IL-6, IL-17A, IP-10, MIP-1α, and TNF-α in sputum were significantly higher in NAEB patients than that in healthy subjects (all *P* < 0.05). Values of area under the receiver operating characteristic curve (AUROC) of these cytokines were all above 0.750. The concentrations of eotaxin and IL-4 were positively correlated with sputum eosinophil percentage (*r* = 0.726, *P* = 0.002; *r* = 0.511, *P* = 0.043; respectively). No significant correlations between other cytokines (EGF, GM-CSF, GRO, IFN-γ, IL-1β, IL-6, IL-17A, IP-10, MIP-1α, and TNF-α) in sputum and sputum eosinophil percentage were found. The level of IL-4 in serum was slightly higher in NAEB patients than in healthy subjects. However, there was no correlation between serum IL-4 levels and sputum eosinophil percentage.

We identified the cytokine profile in sputum and serum from NAEB patients. Sputum eotaxin and IL-4 could have the potential to become the biomarkers for NAEB and might be useful to assist in the diagnosis of NAEB.

## Abstract 2

Supplemental Digital Content is available in the text

## 1 Introduction

Nonasthmatic eosinophilic bronchitis (NAEB) was originally described by Gibson et al in 1989.^[1]^ Characteristics of NAEB patients include chronic cough and airway eosinophilic inflammation. There is no objective evidence of variable airflow obstruction or airway hyperresponsiveness in patients with NAEB.^[2]^ We and other studies have shown that NAEB was a common cause of chronic cough in 10% to 30% of patients among different regions.^[3–7]^

The immunopathology of NAEB is similar to that of asthma. Both these 2 conditions have airway eosinophilic inflammation, reticular lamina, and basement membrane thickening.^[2,8]^ However, Lai et al^[9]^ have found that NAEB could not develop chronic airway obstruction in a prospective, observational study. NAEB is highly likely to a distinct entity, rather than a precursor for asthma. Recently, the assessment of cytokines and inflammatory mediators in airway in NAEB patients has gained attention. Previous studies have found inflammatory molecules, such as cysteinyl leukotrienes, eosinophilic cationic protein, exhaled nitric oxide, 8-isoprostane, and prostaglandin E2 in sputum and bronchioalveolar lavage were elevated in NAEB patients.^[2,10–13]^ Other studies have found the gene expression of interleukin (IL)-5, and granulocyte macrophage colony stimulating factor (GM-CSF) were increased in sputum cells and bronchioalveolar lavage cells in NAEB patients.^[14]^ These studies provide evidence that some inflammatory molecules play a vital role in the development of airway inflammation in NAEB. However, these studies only investigated a small number of inflammatory molecules in airway. It is not clear whether there are other cytokines playing an important role in airway eosinophilic inflammation of NAEB.

In addition to airway inflammation, it is also not yet known whether systemic inflammation involves in the pathogenesis of NAEB. Measurements of many cytokine levels in sputum and serum might be a promising approach to investigate the underling pathologic mechanisms and find potential biomarkers that facilitate the detection of inflammation. Thus, we used the multiplex bead array technology to examine a panel of 21 cytokines and chemokines in sputum and serum from NAEB patients and healthy subjects in this study. Besides, associations between these cytokines and the clinical features of NAEB (such as sputum eosinophil percentage, fractional exhaled nitric oxide (FeNO), and total immunoglobulin E (TIgE)) were evaluated.

## 2 Methods

### 2.1 Subjects

In the cross-sectional study, newly diagnosed and untreated NAEB patients, as well as the healthy subjects, aged 18 to 65 years, were recruited from respiratory outpatient clinics and from staffs in the First Affiliated Hospital of Guangzhou Medical University between October 2014 and January 2016. According to the Chinese cough guideline, NAEB was diagnosed when the patients met the following criteria^[15]^: chronic cough lasting for more than 8 weeks, sputum eosinophilia ≥ 3%, forced expiratory volume in 1 s (FEV_1_) > 80% predicted, FEV_1_/forced vital capacity (FVC) > 80%, and PC20-FEV_1_ (methacholine) > 16 mg/mL, no abnormality in chest radiograph.

Healthy subjects had normal spirometry values and airway responsiveness without a history of allergies, respiratory or systemic diseases, would be recruited. All subjects were nonsmokers. Patients were excluded if they experienced a respiratory infection in the last month, or had a history of bronchiectasis, chronic obstructive pulmonary disease, pulmonary embolism, or other pulmonary diseases. Pregnant or lactating women were also excluded.

All subjects underwent a meticulous clinical assessment, including history-taking, induced sputum, routine blood tests, serum total immunoglobulin E (TIgE) and Phadiatop assays, FeNO, spirometry, and methacholine challenge testing. The study was approved by the Ethics Committee of the First Affiliated Hospital of Guangzhou Medical University. The ID of the ethic approval: 2015-27. Informed consent was obtained from each subject. The study was registered at ClinicalTrials.gov (Registration number: NCT02555345).

### 2.2 Sputum processing

Sputum was induced using inhalations of increasing concentrations (3%, 4%, and 5%) of hypertonic saline via an ultrasonic nebulizer.^[16]^ The sputum sample was mixed with 4 times volume of 0.1% dithiothreitol (DTT) (Sigma Chemicals, Poole, UK) and then filtered through a 48 μm nylon gauze and centrifuged at 3000 rpm for 10 minutes at 4°C. The cell pellet was resuspended in phosphate-buffered saline (PBS) (Sigma Chemicals). The cell smear was prepared and stained with hematoxylin–eosin stain. The differential cell counts of sputum samples were obtained by counting 400 nonsquamous cells. The supernatant was split into 200 μL aliquots and frozen at −80°C for subsequent assay of cytokines. Sputum samples containing more than 10% squamous cells were excluded from the analysis.

### 2.3 FeNO assessment

Measurement of the FeNO was performed using the NIOX MINO instrument (Aerocrine Co. Ltd, Solna, Sweden) in accordance with the recommendations of international guidelines.^[17]^ Briefly, subjects were informed to take a deep inhalation of the gases free of nitric oxide to the total lung capacity through a mouthpiece, followed by the exhalation at a constant flow (50 mL/s) for 10 seconds.

### 2.4 Spirometry and bronchial provocation test

Spirometry and bronchial provocation test were conducted using Masterscreen spirometers (Jaeger Co. Ltd, Hochberg, Germany) according to the American Thoracic Society Recommendation.^[18]^ Lung function parameters including FEV_1_% predicted and FEV_1_/FVC ratio were recorded. Airway hyperresponsiveness was defined as a ≥20% decrease in the FEV_1_ at a methacholine dose of 12.8 μmol or less.

### 2.5 Peripheral blood eosinophil counts

Peripheral venous blood samples were collected in ethylenediamineteraacetic acid (EDTA) tubes. An automated analyzer (Beckman Coulter, Miami, FL) was used to determine the eosinophil counts.

### 2.6 Serum samples and immunoglobulin E measurement

Peripheral venous blood was collected in coagulation-promoting tubes. Serum was obtained after centrifugation at 3000 rpm for 10 minutes at 4°C and then aliquoted and stored at −80°C until further analysis. Serum levels of total IgE and specific IgE were measured with ImmunoCAP 100 (Pharmacia Diagnostics, Uppsala, Sweden) according to the manufacturer's instructions. The Phadiatop test included the following allergens: Artemisia and mixed trees (Acer, Acacia, Betula, Eucalyptus, Malaleuca, Olea, Pinus, Quercus, Salix, and Ulmus), mites (*Dermatophagoides pteronyssinus* and *D. fariane*), mixed grasses (Cynodon, Lolium, Parietaria, and Phleum), mixed moulds (Alternaria, Aspergillus, Cladosporium, and Penicillium), and pets (cat and dog). With a cutoff value of 0.35 kU/L, the Phadiatop test with the specific IgE level of over 0.35 kU/L was defined as atopy-positive.

### 2.7 Multiplex cytokine assay

A bead-based multiply cytokine assay kit (HCYTOMAG-60k, Merck Millipore, Darmstadt, Germany) was used to determine the cytokine profile in sputum and serum including following cytokines/chemokines: epidermal growth factor (EGF), eotaxin/CCL11, fibroblast growth factor 2 (FGF2), granulocyte-macrophage colony-stimulating factor (GM-CSF), growth-regulated oncogene (GRO)/CXCL1, interferon (IFN)-α2, IFN-γ, IL-1β, IL-2, IL-4, IL-5, IL-6, IL-8, IL-13, IL-17A, interferon gamma-induced protein (IP)-10/CXCL10, monocyte chemotactic protein (MCP)-1/CCL2, macrophage inflammatory protein (MIP)-1α/CCL3, MIP-1β/CCL4, tumor necrosis factor (TNF)-α, and vascular endothelial growth factor (VEGF)-A.

### 2.8 Statistical analysis

Statistical analyses were performed with SPSS 19.0 (SPSS, Inc., Chicago, IL). The values were presented as mean ± SD or median (range) for continuous variables, and as percentage for categorical variables. Two samples independent *T* test was performed for normally distributed data in the 2-group comparisons. The Mann–Whitney *U* test was performed for abnormally distributed data in the 2-group comparisons. Unbiased/unsupervised agglomerative (“bottoms-up”) hierarchical clustering was performed on Z normalized data by using the uncentered correlation as the similarity metric (Cluster version 2.11, Eisen Lab, Stanford, California, USA). The dendrogram and resulting heatmap were visualized using TreeView (version 1.60, Eisen Lab). The MedCalc software (MedCalc, Mariakerke, Belgium) was used to obtain the receiver-operating characteristic (ROC) curves. Area under the ROC curve (AUROC) was used to evaluate the ability of each cytokine level to discriminate NAEB patients from healthy subjects. To assess the relationship between and within cytokines and clinical indices of NAEB, Pearson or Spearman rank-correlation test was performed. Two-tailed *P* values of <0.05 were considered to indicate statistical significance.

## 3 Results

### 3.1 Demography of patients and healthy subjects

**Figure 1:**
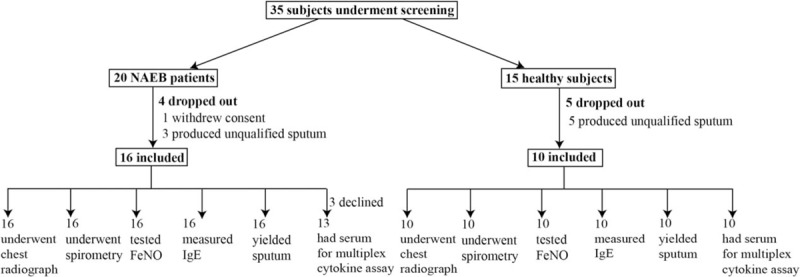
Subject recruitment flowchart.

The recruitment process is outlined in Fig. 1. We enrolled 16 NAEB patients and 10 healthy subjects. Clinical characteristics of subjects are described in Table 1. The NAEB patients had significantly higher levels of blood eosinophil counts, FeNO, sputum eosinophil percentage, and TIgE than the healthy subjects (all *P* < 0.05). The NAEB group had lower levels of sputum macrophage percentage than the healthy subjects (*P* < 0.05). There were no differences with respect to gender, age, BMI, FEV_1_%predicted, FEV_1_%FVC, sputum neutrophil percentage, and sputum lymphocyte percentage between 2 groups (all *P* > 0.05).

**Table 1:**
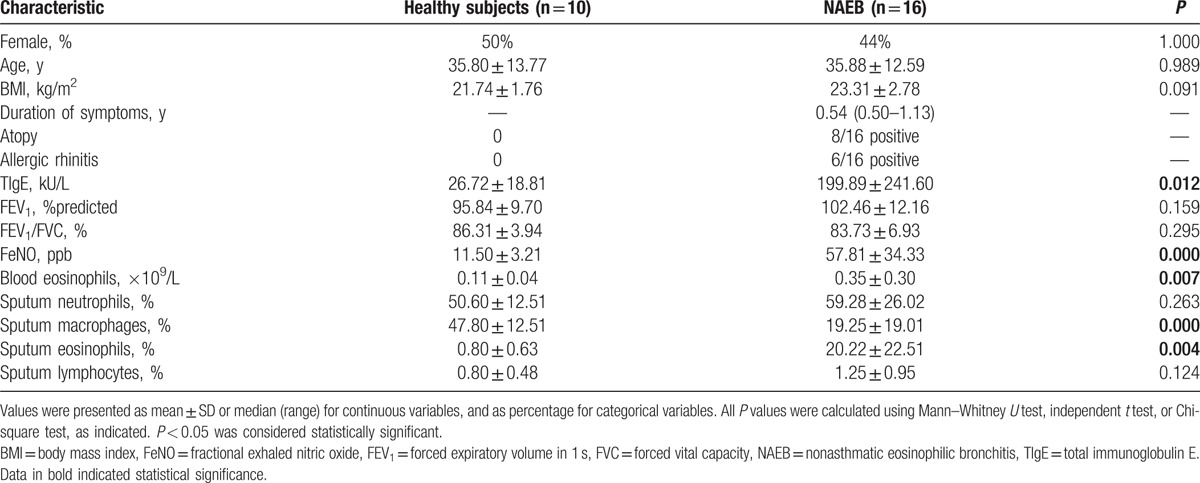
Clinical characteristics of NAEB patients and healthy subjects.

### 3.2 Sputum cytokine profile in NAEB patients and healthy subjects

**Figure 2:**
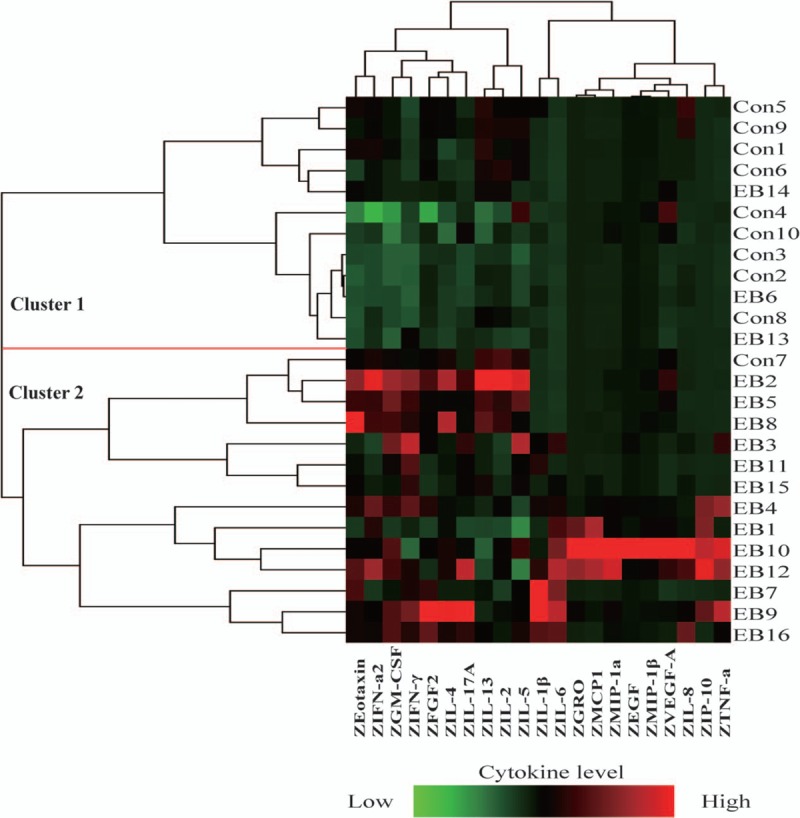
Heatmap depicting hierarchical clustering of sputum cytokine profile. Twenty-one cytokine levels in 16 patients with NAEB and 10 healthy subjects were shown. Each row is an individual subject. Each column is a cytokine. Left, dendogram shows the similarity of the samples. Right, numbers are the subject numbers.

Hierarchical clustering of the sputum cytokine profile in patients with NAEB and healthy subjects is shown in Fig. 2. Most of patients with NAEB (n = 13) were grouped together in the second major branch of the dendrogram on the basis of the levels of 21 sputum cytokines (cluster 2). However, 3 patients with NAEB were indistinguishable from healthy subjects. This subset of patients with NAEB and healthy subjects (n = 9) were grouped together in the first major branch of the dendrogram (cluster 1).

The results of comparison analysis of the sputum cytokine levels in 2 groups are summarized in Table 2. Sputum cytokine profile in NAEB group showed the increased levels of EGF, eotaxin, GM-CSF, GRO, IFN-γ, IL-1β, IL-4, IL-6, IL-17A, IP-10, MIP-1α, and TNF-α compared with healthy subjects (all *P* < 0.05). However, no significant differences were detected between NAEB patients and healthy subjects in the levels of FGF2, IFN-α2, IL-2, IL-5, IL-8, IL-13, MCP-1, MIP-1β, and VEGF-A (all *P* > 0.05).

**Table 2:**
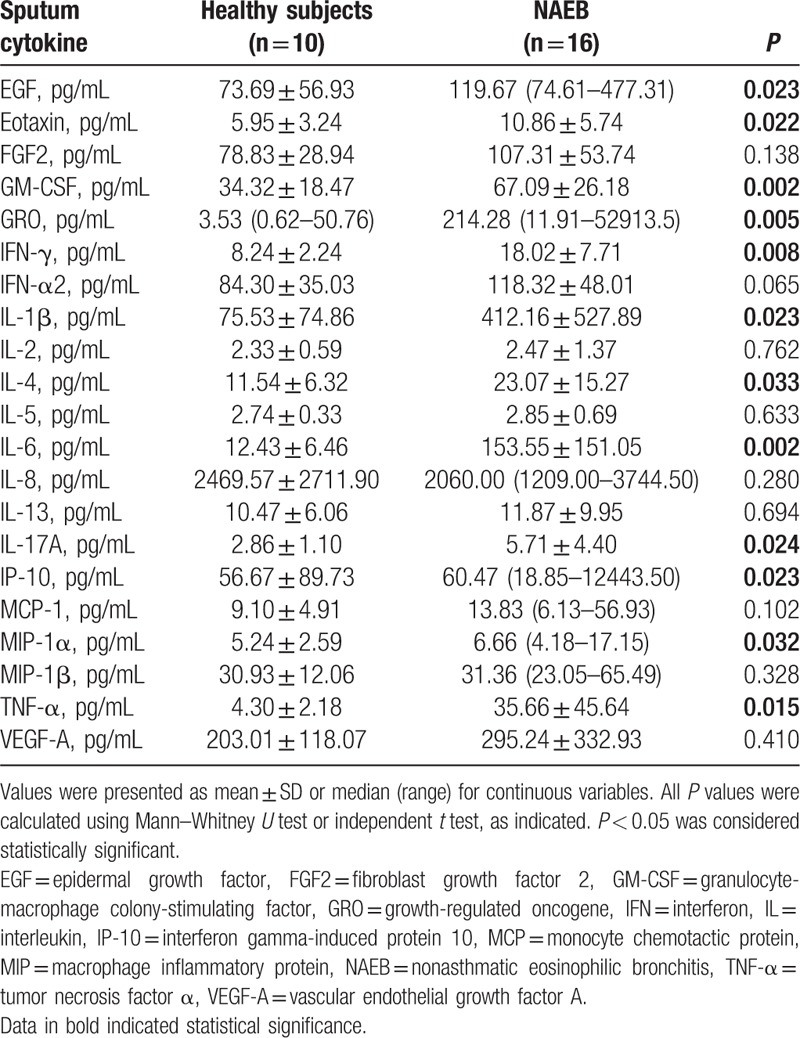
Sputum cytokine levels in NAEB patients versus healthy subjects.

### 3.3 Sputum cytokines distinguish patients with NAEB from healthy subjects

The AUROC for all 12 increased cytokines (EGF, eotaxin, GM-CSF, GRO, IFN-γ, IL-1β, IL-4, IL-6, IL-17A, IP-10, MIP-1α, and TNF-α) in sputum is presented in Table S1. These cytokines had area under the receiver operating characteristic curve (AUROC) values of above 0.750, which were capable for distinguishing patients with NAEB from healthy subjects.

### 3.4 Associations between and within sputum cytokines and clinical indices of NAEB patients

Correlations between sputum cytokines and sputum eosinophil percentage in NAEB patients are shown in Table 3. There was a significant correlation between eotaxin level and sputum eosinophil percentage (*r* = 0.726; *P* = 0.002) (Fig. S1A). A similar correlation was observed between IL-4 level and sputum eosinophil percentage (*r* = 0.511; *P* = 0.043) (Fig. S1B). In contrast with these results, no significant correlations between other cytokines (EGF, GM-CSF, GRO, IFN-γ, IL-1β, IL-6, IL-17A, IP-10, MIP-1α, and TNF-α) and sputum eosinophil percentage were found. In addition, we also assessed the correlations between all 12 increased cytokines in sputum and FeNO, and TIgE. However, there was not any relationship between cytokines and FeNO, and TIgE (all *P* > 0.05).

**Table 3:**
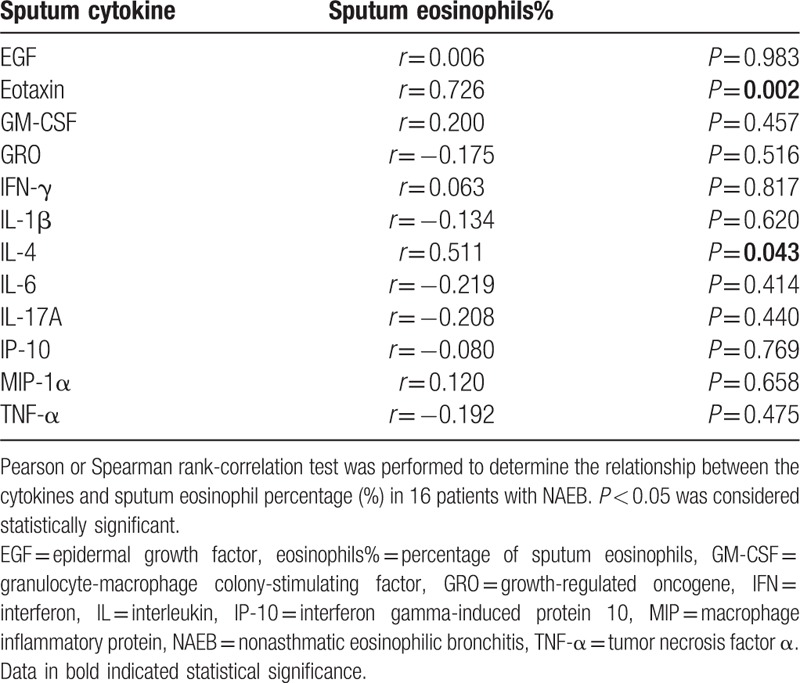
Correlation between the sputum cytokines and sputum eosinophil percentage in NAEB patients.

Spearman correlations between sputum cytokines (EGF, eotaxin, GM-CSF, GRO, IFN-γ, IL-1β, IL-4, IL-6, IL-17A, IP-10, MIP-1α, and TNF-α) in NAEB patients are presented in Table S2. IL-4 was significantly positively correlated with eotaxin (*r* = 0.661, *P* = 0.005), GM-CSF (*r* = 0.705, *P* = 0.002), and Th17A (*r* = 0.574, *P* = 0.020). A number of other significant correlations between sputum cytokines (e.g., TNF-α was also significantly associated with other cytokines) were also observed.

### 3.5 Systemic inflammation

The results of serum cytokine levels in 2 groups are shown in Table 4. The levels of IL-4 in serum were slightly higher in NAEB patients than in the healthy subjects (*P* = 0.027). However, there were no significant correlations between the serum IL-4 levels and sputum eosinophil percentage, FeNO, and TIgE in NAEB patients.

**Table 4:**
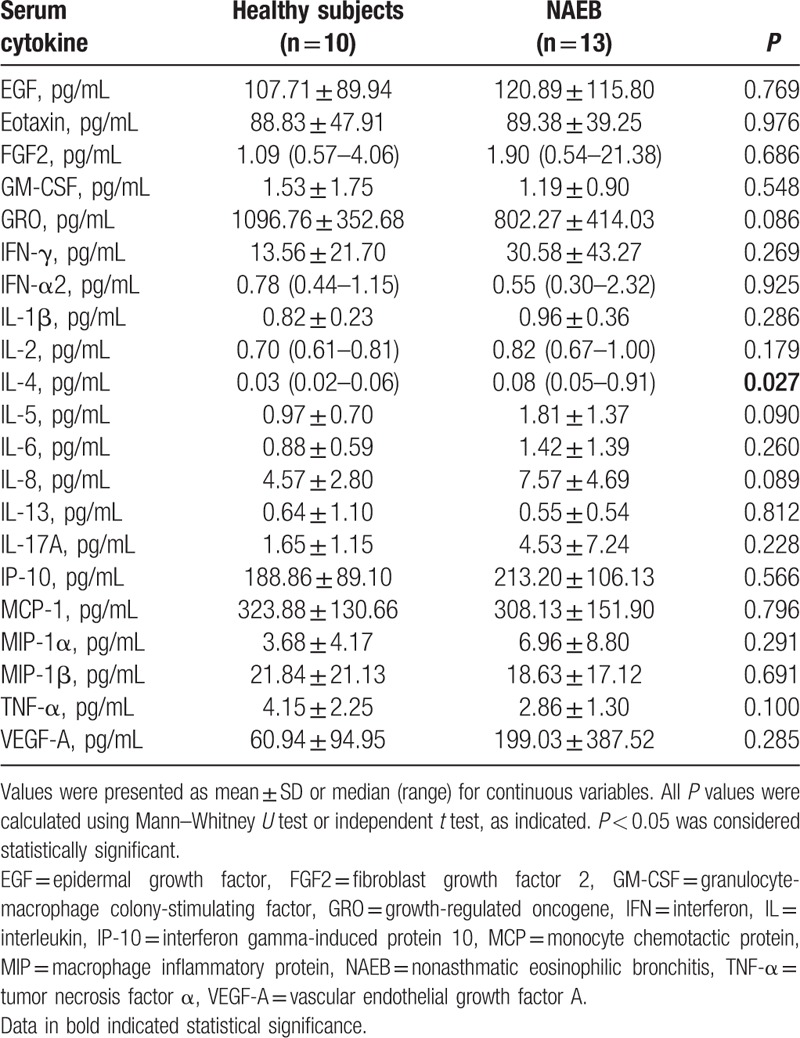
Serum cytokine levels in NAEB patients and healthy subjects.

## 4 Discussion

In this study, a significant increase in 12 out of the 21 cytokines/chemokines was observed in sputum in NAEB patients compared with the healthy subjects. These cytokines/chemokines included EGF, eotaxin, GM-CSF, GRO, IFN-γ, IL-1β, IL-4, IL-6, IL-17A, IP-10, MIP-1α, and TNF-α that exhibited good performance. However, in serum, only the IL-4 level was slightly higher in NAEB patients than in the healthy subjects. This indicated that airway inflammation, but not systemic inflammation, might play the main pathologic role in NAEB.

We found T helper (Th) 1 cytokines (IFN-γ, TNF-α, and GM-CSF), and Th17 cytokines (IL-1β, IL-6, and IL-17A) in sputum were increased in NAEB patients. The upregulation of IFN-γ in airway of NAEB patients was in agreement with the findings of previous studies on brochoalveolar lavage fluid and mucosal biopsy.^[19]^ Th1 and Th17 cytokines could recruit neutrophils to local sites of inflammation and may mediate airway neutrophilic inflammation.^[20–23]^ In our study, about 40% of NAEB patients presented a mixed eosinophilic/neutrophilic subtype (eosinophil ≥ 3.0%, neutrophil ≥ 61%). Up to date, the mechanistic contribution of neutrophils to NAEB is not clear. Our results suggested that Th1 and Th17 cytokines might mediate airway neutrophilic inflammation in certain group of NAEB patients. Further studies are required to fully assess the contribution of Th1 and Th17 cytokines to airway neutrophilic inflammation of NAEB. In addition, we found EGF, GRO (a neutrophil chemoattractant), IP-10 (both Th1 and Th2 chemokine) and MIP1-α (a weak eosinophil chemoattractant), in sputum were increased in NAEB patients, which indicated these cytokines might play a potential role in the pathophysiology of NAEB.

In the present study, IL-4 levels were increased in induced sputum, and significantly correlated with sputum eosinophils and sputum eotaxin levels in patients with NAEB. Generally, NAEB is regarded as a Th2-driven disease.^[19]^ IL-4 is a Th2 cytokine that contributes to activating and recruiting of eosinophils, IgE antibody-producing B cells, and airway mucus production.^[23–26]^ IL-5 is also the Th2 cytokine that is essential for the survival, differentiation, proliferation, maturation, recruitment, and activation of eosinophils.^[27]^ Our results demonstrated that IL-4 could be involved in airway eosinophilic inflammation of NAEB. However, it is somewhat surprising that IL-5 was not elevated in sputum from NAEB patients in our study. Clinical studies have shown that IL-5 was associated with airway diseases with airway eosinophilic inflammation, particularly asthma and NAEB, and IL-5 was related to the severity of asthma.^[13,14,28]^ Three reasons might account for this. First, the intensity of airway inflammation in patients with mild NAEB was low in the present study. Second, our study was performed on unconcentrated sputum using multiplex beads, which might lead to the low concentrations of IL-5. Third, possible effects of DTT on IL-5 measurement could not be completely excluded.^[29]^

In our study, sputum eotaxin levels were significantly correlated with sputum eosinophilic percentage. Eotaxin belongs to the CC family of chemokines, which are predominantly chemotactic for inflammatory cells. It is mainly produced by epithelial cells, but in some conditions it is also produced by alveolar macrophages, and mast cells.^[30,31]^ Eotaxin mainly induces chemotaxis and migration of eosinophils to certain tissues via interaction with CC chemokine receptor (CCR) 3 which is expressed on eosinophils.^[32–36]^ It is known that eosinophil accumulation in the airway is a prominent feature of NAEB. Our study found that the sputum eotaxin, but not serum eotaxin, could be involved in eosinophil recruitment into the airway in NAEB patients.

Moreover, our data have shown that a very small percentage of patients with NAEB could have lower cytokine levels, despite NAEB patients with sputum eosinophils over 3%. The cause of this remains obscure, but possibility includes a very mild condition, and intrinsic defects in barrier function.

Some limitations of this study should be considered. First, the sample size would be small in the present study. There was no previous data representing a similar population that could be used to estimate standard deviations of the groups, and therefore a priori power calculation was not performed. In the further study, a priori sample size calculation would be performed based on this study and larger sample sizes are required to externally validate our findings. Second, we did find sputum eotaxin and IL-4 increased in NAEB patients and correlated with sputum eosinophils. It should be recognized that the low levels of eotaxin and IL-4 might be secreted by airway epithelium. This indicates that high sensitivity of protein assays could be required for the future quantitation of eotaxin and IL-4. Third, we only observed the baseline, but did not include the change of cytokines/chemokines in NAEB patients after treatment. Further studies are required, involving samples from different populations of patients, as well as the follow up in order to understand how the cytokine levels change after treatment and whether these cytokines can predict the response to treatment. In addition, better understanding of how the various cytokines interact to influence the airway inflammation of NAEB might have important implications for understanding the pathogenesis of NAEB. Finally, we focused on NAEB patients and healthy controls, and did not compare the cytokine levels in sputum and serum with relation to other airway diseases (such as asthma or lung eosinophilia with airflow obstruction). Therefore, we could not differentiate NAEB from other airway diseases based on the spectrum of cytokines. Whether the cytokines could be applied to differentiate NAEB from other airway diseases requires further study.

In conclusion, we identified the cytokine profile in sputum and serum from NAEB patients. Eotaxin and IL-4 levels in sputum could distinguish patients with NAEB from healthy subjects, and both correlated positively with sputum eosinophil percentage. This indicates eotaxin and IL-4 in sputum might play a role in the pathogenesis of NAEB and could have the potential to become the biomarkers for NAEB, and might be useful to assist in the diagnosis of NAEB.

## Acknowledgments

We thank professor Ting Luo, MD (Guangdong Laboratory Animals Monitoring Institute) for technical assistance on experiment of multiplex cytokine assay. Special thanks are given to Mei Jiang, MD, PhD (State Key Laboratory of Respiratory Disease, Guangzhou Institute of Respiratory Disease, First Affiliated Hospital of Guangzhou Medical University, China) for data analysis.

## Supplementary Material

Supplemental Digital Content
