# Supplementary material for: Eotaxin and IL-4 levels are increased in induced sputum and correlate with sputum eosinophils in patients with nonasthmatic eosinophilic bronchitis
Source: Medicine (Baltimore). 2017 Mar 31;96(13):e6492. doi: 10.1097/MD.0000000000006492 (PMC5380279; doi:10.1097/MD.0000000000006492)

**Table S1** Predictive value of sputum cytokines for patients with NAEB

| **Cytokine** | **AUROC** | **95%CI** | **P** | **Yoden J** | **Sensitivity** | **Specificity** |
| --- | --- | --- | --- | --- | --- | --- |
| **EGF** | 0.769 | 0.563-0.910 | 0.006 | 0.488 | 68.75 | 80.00 |
| **eotaxin** | 0.781 | 0.577-0.918 | 0.003 | 0.475 | 87.50 | 60.00 |
| **GM-CSF** | 0.822 | 0.623-0.943 | <0.001 | 0.563 | 56.25 | 100.00 |
| **GRO** | 0.831 | 0.634-0.948 | <0.001 | 0.638 | 93.75 | 70.00 |
| **IFN-γ** | 0.866 | 0.675-0.967 | <0.001 | 0.775 | 87.50 | 90.00 |
| **IL-1β** | 0.813 | 0.612-0.937 | <0.001 | 0.613 | 81.25 | 80.00 |
| **IL-4** | 0.762 | 0.556-0.906 | 0.012 | 0.575 | 87.50 | 70.00 |
| **IL-6** | 0.844 | 0.648-0.955 | <0.001 | 0.625 | 62.50 | 100.00 |
| **IL-17A** | 0.794 | 0.591-0.926 | 0.002 | 0.588 | 68.75 | 90.00 |
| **IP-10** | 0.769 | 0.563-0.910 | 0.008 | 0.538 | 93.75 | 60.00 |
| **MIP-1α** | 0.753 | 0.546-0.900 | 0.011 | 0.400 | 100.00 | 40.00 |
| **TNF-α** | 0.844 | 0.648-0.955 | <0.001 | 0.613 | 81.25 | 80.00 |

AUROC, area under the receive operating characteristic curve; CI: confidence interval; EGF, epidermal growth factor; GM-CSF, granulocyte-macrophage colony-stimulating factor; GRO, growth regulated oncogene; IFN, interferon; IL, interleukin; IP-10, interferon gamma-induced protein 10; MIP, macrophage inflammatory protein; NAEB, nonasthmatic eosinophilic bronchitis; TNF-α, tumor necrosis factor α.

**Table S2** Spearman correlations between sputum cytokines in NAEB patients

| **Cytokine** | **EGF** | | **eotaxin** | **GM-CSF** | **IFN-γ** | **GRO** | **IL-17A** | **IL-1β** | **IL-4** | **IL-6** | **IP-10** | **MIP-1α** | **TNF-α** |
| --- | --- | --- | --- | --- | --- | --- | --- | --- | --- | --- | --- | --- | --- |
| **EGF** | r | 1.000 | 0.024 | 0.155 | -0.056 | **0.888***** | 0.224 | **0.568*** | 0.195 | **0.779***** | **0.830***** | **0.685**** | **0.885***** |
| **eotaxin** | r |  | 1.000 | **0.570*** | 0.348 | -0.164 | 0.471 | -0.041 | **0.661**** | 0.258 | 0.161 | 0.432 | 0.171 |
| **GM-CSF** | r |  |  | 1.000 | **0.629**** | 0.004 | **0.757**** | 0.025 | **0.705**** | 0.294 | 0.357 | **0.567*** | 0.389 |
| **IFN-γ** | r |  |  |  | 1.000 | -0.118 | **0.691**** | 0.127 | 0.313 | 0.112 | 0.161 | 0.130 | 0.217 |
| **GRO** | r |  |  |  |  | 1.000 | 0.194 | **0.600*** | 0.135 | **0.803***** | **0.882***** | **0.666**** | **0.876***** |
| **IL-17A** | r |  |  |  |  |  | 1.000 | 0.341 | **0.574*** | 0.462 | 0.432 | 0.428 | 0.462 |
| **IL-1β** | r |  |  |  |  |  |  | 1.000 | 0.007 | **0.750**** | 0.416 | 0.335 | **0.624*** |
| **IL-4** | r |  |  |  |  |  |  |  | 1.00 | 0.401 | 0.432 | **0.717**** | 0.418 |
| **IL-6** | r |  |  |  |  |  |  |  |  | 1.000 | **0.801***** | **0.738**** | **0.865***** |
| **IP-10** | r |  |  |  |  |  |  |  |  |  | 1.000 | **0.846***** | **0.898***** |
| **MIP-1α** | r |  |  |  |  |  |  |  |  |  |  | 1.000 | **0.830***** |
| **TNF-α** | r |  |  |  |  |  |  |  |  |  |  |  | 1.000 |

*****P < 0.05; ******P <0.01; *******P <0.001. EGF, epidermal growth factor; GM-CSF, granulocyte-macrophage colony-stimulating factor; GRO, growth regulated oncogene; IFN, interferon; IL, interleukin; IP-10, interferon gamma-induced protein 10; MIP, macrophage inflammatory protein; NAEB, nonasthmatic eosinophilic bronchitis; TNF-α, tumor necrosis factor α.

**Figure S1** Correlation between the levels of eotaxin and IL-4 and percentage of sputum eosinophils (%) in patients with NAEB. (A) Correlation of eotaxin level and percentage of sputum eosinophils (%) was found in 16 patients with NAEB (r░=░0.726; P░=░0.002); (B) Correlation of IL-4 level and percentage of sputum eosinophils (%) was also found in 16 patients with NAEB (r░=░0.511; P░=░0.043).


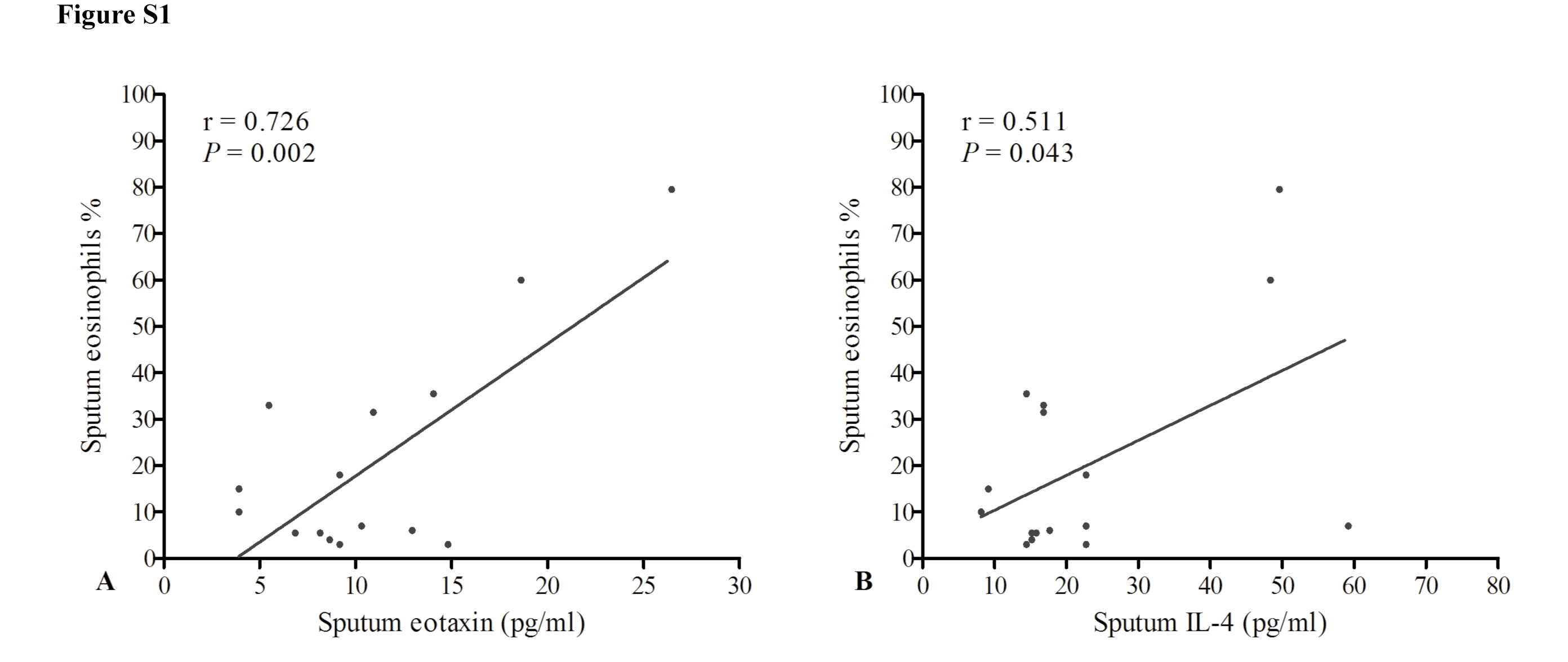

Supplement: Supplemental Digital Content [file medi-96-e6492-s001.doc]
